# MicroRNA Expression Profiling Screen miR-3557/324-Targeted* CaMK/mTOR* in the Rat Striatum of Parkinson's Disease in Regular Aerobic Exercise

**DOI:** 10.1155/2019/7654798

**Published:** 2019-06-12

**Authors:** Wenfeng Liu, Li Li, Shaopeng Liu, Zhiyuan Wang, Heyu Kuang, Yan Xia, Changfa Tang, Dazhong Yin

**Affiliations:** ^1^Hunan Provincial Key Laboratory of Physical Fitness and Sports Rehabilitation, Hunan Normal University, Changsha, Hunan, 410012, China; ^2^Department of Experimental and Clinical Pharmacology, University of Minnesota, Minneapolis, MN 55455, USA; ^3^The Key Laboratory of Protein Chemistry and Developmental Biology of Ministry of Education, Hunan Normal University, Changsha, Hunan, 410081, China; ^4^School of Health & Kinesiology, Georgia Southern University, Statesboro, GA 30460, USA; ^5^Qingyuan People's Hospital, The Sixth Affiliated Hospital, Guangzhou Medical University, Guangzhou, Guangdong, 511500, China

## Abstract

This study aimed to screen the target miRNAs and to investigate the differential miR-3557/324-targeted signal mechanisms in the rats' model of Parkinson's disease (PD) with regular aerobic exercise. Rats were divided into sedentary control PD group (SED-PD,* n* = 18) and aerobic exercise PD group (EX-PD,* n* = 22). After 8 weeks of regular aerobic exercise, a 6-hydroxydopamine- (6-OHDA-) induced PD lesion model was constructed. Preregular aerobic exercises enhanced the injury resistance of rats with 6-OHDA-induced PD. The rotational behavior after injection of apomorphine hydrochloride was alleviated. Under the scanning electron microscopy, we found the neurons, axons, and villi of the striatum were clearly and tightly arranged, and neurons and axons significantly becoming larger. Tyrosine hydroxylase (TH) was increased significantly and *α*-synuclein protein expression was reduced in the EX-PD group compared to the SED-PD group. Screening from miRNA microarray chip, we further found upregulation of miR-3557 and downregulation of miR-324 were closely related to the calcium-modulating signaling pathway, remitting the progress of Parkinson's disease on aerobic exercise. Compared to the SED-PD group, Ca^2+^/calmodulin dependent protein kinase II (*CaMK2α*) was upregulated, but* CaMKV* and voltage-dependent anion-selective channel protein 1 (*Vdac1*) were significantly downregulated in the EX-PD group. Additionally, phosphatidylinositol-3-kinase (*PI3K*)/mammalian target of rapamycin (*mTOR*) expression were activated, and ubiquitin carboxy-terminal hydrolase L1 (*UCH-L1*) expression was upregulated in the EX-PD group. Conclusions: the adaptive mechanism of regular aerobic exercise delaying neurodegenerative diseases and lesions was that miR-3557/324 was activated to regulate one of its targets CaMKs signaling pathways. CaMKs, coordinated with mTOR pathway-related gene expression, improved UCH-L1 level to favor for delaying neurodegeneration or improving the pathogenesis of PD lesions.

## 1. Introduction

microRNAs (miRNAs or miR) are associated with various diseases, such as neurodegenerative diseases [Alzheimer's disease (AD), Parkinson's disease (PD), and Schizophrenia] [1], cardiovascular diseases (myocardial infarction, myocardial fibrosis, and atherosclerosis) [2], and metabolic diseases (diabetes and obesity) [3]. In the present study on the miRNAs, about 70% miRNAs were expressed in the brain tissue of mammals, such as specific miR-124a, miRNA-128, and miRNA-101. miRNAs were rich in brain tissues (such as miR-125b) and important in the regulation of gene expression in different physiological processes and signaling pathways of the nervous system, such as the development and progression of the nervous system, neural stem cell differentiation, and apoptosis [4]. Liu et al. [5] reported that* Drosophila melanogaster* began to express miR-34 in its brain in the adulthood and that if miR-34 was missing, protein misfolding increased, neurodegenerative diseases of the brain occurred, and the life expectancy was shortened.

In addition, miRNAs may also participate in the occurrence of neurodegenerative diseases through some specific molecular mechanisms. Nelson et al. [6] found that miR-25 downregulated the expression of synaptic proteins in the brain of patients with AD and synergized with activated reactive oxygen species (ROS). Wang et al. [7] reported that miR-34a could induce neuronal apoptosis by inhibiting B-cell lymphoma 2 (Bcl-2) in neuronal development and senescence and that miR-34a was expressed in patients with AD. Lemaitre et al. [8] showed that the expression of miR-433 decreased and the expression of fibroblast growth factor 20 (*FGF20*) increased in patients with PD, while* FGF20* promoted the expression of *α*-synuclein, which is the most critical protein in PD pathogenesis. Packer et al. [9] found that the expression of miR-9 decreased in patients with PD patients, which might be involved in the pathogenesis of PD by modulating the target gene RE1-Silencing Transcription factor (REST)/coREST. miR-133 regulated the expression of transcription factor N-myc downregulated gene 1 (*NGRG1*) associated with PD; miR-7, miR-153, and miR-433 regulated the expression of *α*-synuclein in PD [10]. Dostie et al. [11] showed that miR-175 was associated with early clinical manifestations of PD. miR-132 and miR-212 were involved in the occurrence of various abnormalities, such as synaptic plasticity and connectivity in schizophrenia [12].

Yet, no known study has examined how the miRNAs (examined via miRNAs profiling) might differ between PD animals who* regularly* exercise interventions vs. PD animals who are* habitually* sedentary. A study of this type is needed to discern whether miRNAs can be employed as a biomarker of neuronal functional decline and whether regular exercise may more greatly attenuate neurodegeneration. The purpose of the exercise-adaptive stress-reversing exercise was to seek more direct evidence of regulating mRNA in related pathways, thereby identifying the biological functions of genes that regulated the key interpretable motor-adapted mechanisms of action. We found the adaptive mechanism of regular aerobic exercise delaying the development of neurodegenerative diseases and lesions was to initiate miR-3557 and miR-324 to regulate one of its target Ca^2+^/calmodulin dependent protein kinase (CaMKs) signaling pathways and to coordinate with regulating closely related phosphatidylinositol-3-kinase (PI3K)/ protein kinase B(Akt) /mammalian target of rapamycin (mTOR) pathway protein expression, hence delaying the progression of neurodegeneration or alleviating the development of PD lesions.

## 2. Materials and Methods

### 2.1. Experimental Animals, Ethics Statement, and Exercise Protocol

#### 2.1.1. Experimental Animals and Ethics Statement

Specific-pathogen-free 13-month-old male Sprague–Dawley rats (*n* = 40, 549.50±16.13 g) were supplied by the Animal Center of East Biotechnology Services Company (Changsha, Hunan, China; License number: Xiang SCXK 2009-0012). Four or five rats were housed in one standard cage (three or four rats/cage) with free access to food and water. All animals were kept in an air-conditioned room maintained at a constant temperature of 20°C–25°C with a relative humidity of 45%–55%. They were subjected to a cycle of 12-h light and 12-h darkness. They were acclimated to laboratory conditions for 1 week, prior to the start of the experiment. A total of 40 rats were randomly assigned to 2 groups: sedentary control PD group (SED-PD,* n* = 18) and aerobic exercise PD group (EX-PD,* n* = 22). After 8 weeks of aerobic exercise in EX-PD, the SED-PD and EX-PD groups were administered a unilateral injection of 6-OHDA and the no-lesion side (N) and the lesion side (L) were formed, respectively.

All animal procedures were approved by the local ethics committee (the Institutional Review Board of Hunan Normal University) and the Guidelines for Care and Use of Laboratory Animals (Washington DC 2011). Disposal of animals was done in accordance with* The Guidance on the Care of Laboratory Animals*.” (The provisions were issued in 2006 by the Ministry of Science and Technology of the People's Republic of China.)

#### 2.1.2. Exercise Protocol

Recently, da Costa and Tuon et al. [13, 14] corroborated that aerobic treadmill exercise before unilateral intrastriatal 6-OHDA injection rescued motor deficits, measured by the apomorphine-induced rotational asymmetry in rats. We performed regular aerobic exercise before unilateral intrastriatal 6-OHDA injection in accordance with newfangled researches [13, 15–17] and exercise load standards reference to Bedford et al. [18]. All animals in the SED-PD and EX-PD groups were first subjected to a 5-day adaptation period on a rat treadmill (slope gradient 0%, ZH-PT-1 Treadmill, Li Tai Bio-Equipment Co., Ltd, Hangzhou, Zhejiang, China). Adapted training was carried out at a speed of 10 m/min and a gradient of 0°, for a gradually increasing duration of time: 10 min on the 1st day, 20 min on the 2nd day, and 25 min on the 3rd-5th day. During this period, they were placed on a belt facing away from the electrified grid (0.6-mA intensity) once per day.

For the actual experiment, SED-PD was in habitual sedentary behavior, but the EX-PD group underwent daily training by running at 15 m/min, equivalent to approx. 50%–60% maximum oxygen consumption ($\dot{V}$O2max) [18, 19], on a slope of 0° for a duration of 15 min. During the first week at the start of the training, the exercise duration was increased gradually from 15 min to 20 min in the second week, 25 min in the third week, 30 min in the fourth week, and 35 min in the fifth and sixth weeks. The EX-PD group underwent daily training by running at 20 m/min and maintained speed for a week (equivalent to approx. 65%–70% $\dot{V}$O2max) [18] on a slope of 0° for a duration of 40 min in the seventh to the eighth weeks. The acceleration of the treadmill was set such that the final speed of 22 m/min was achieved in about 3 min after the start of the training. Sound stimulation and a small wooden stick were used to stimulate the animals' tails, when necessary, to ensure that the animals completed the exercise regime. Also, electrical stimulation (0.6 mA intensity) was used to keep the rats at a one-third distance on the treadmill runway. The rats were required to perform the training 5 days a week for a total of 8 weeks.

### 2.2. Model of PD Induced by 6-OHDA in Rats

All surgical procedures were completed and performed under anesthesia, injected intraperitoneally with 10% chloral hydrate at 3 mL/kg body weight. All efforts were made to minimize the rats' suffering and distress. The EX-PD group fasted for 24 h after eight weeks of regular aerobic exercise, and the rats in SED-PD were carried out in the same way after eight weeks of habitual sedentary behavior. After anesthesia, the brains of the rats were fixed on the brain stereotaxic instrument. The skin was cut and treated with sterilization to expose the anterior skull, and the intersection between the anterior ridge and the midline was determined to be zero in coordinates. Referring to the George Paxinos and Charles Watson's original (Zhuge Qiyin) rat brain stereotaxic map, the right striatum dual coordinates were determined: the first target, 1.0 mm anterior to the anterior ridge, 3.0 mm to the right of the midline, 7.0 mm subdurally; the second target, 0.2 mm posterior to the anterior ridge, 2.6 mm to the right of the midline, and 6.0 mm subdurally. Triangular needles were labeled, and the wells were drilled. Each well was pipetted with 5.0 *μ*L of 6-hydroxydopamine (6-OHDA, total 40*μ*L, 4.0 *μ*g/*μ*L) to inject the aforementioned two targets. 6-OHDA was dissolved in 0.9% sodium chloride, and 0.2 mg/mL ascorbic acid was dissolved in 0.9% sodium chloride. The injection rate was 0.50 *μ*L/min. Each point was injected for 10 min. After the injection, the needle was retained for 5 min and then withdrawn at a speed of 1.0 mm/min. After the injection, the wound was sealed with bone wax, the head skin was sutured, and the local skin was routinely disinfected. At the same time, each rat was injected with clindamycin intraperitoneally twice a week to prevent infection, and the cage was normally reared. Behavioral rotation test, tyrosine hydroxylase (TH), and *α*-synuclein was detected to test the PD model induced by 6-OHDA in rats.

### 2.3. Behavioral Identification on Rat PD Model

In the first postoperative week, the rats were placed in a plastic circular tube with a diameter of about 28 cm and a height of 25 cm or more. Before each rat was placed, alcohol was disinfected and deodorized, and then 0.50 mg/kg body weight of rats was given using apomorphine hydrochloride. The intraperitoneal injection was used to induce the rotation to the contralateral side, and the number of rotations of the rat to the contralateral side was recorded. Each measurement lasted 40 min. The number of rotations in the first-10min, second-10min, and third-10min were recorded, once per week; more than seven rotations per minute was the success model [20].

### 2.4. Tissue Specimen Collection and Analysis

The rats were anesthetized with chloral hydrate (400 mg/kg, intraperitoneally) and decapitated in two weeks after 6-OHDA injection and lesion maturation [21, 22]. The striatum (*Stereotaxic Atlas of the Rat Brain*; George Paxinos., 2005) was dissected from all rats. Tissue samples were stored at −80°C until ready to be used for miRCURY LNA miRNA Array and real-time fluorescence quantitative PCR. All surgical procedures were performed under anesthesia induced using chloral hydrate. All efforts were made to minimize suffering and distress in animals. The striatum tissue was quickly separated. The tissue block was cut into cubes of 3 × 3 mm^2^ with a razor blade, placed in a precooled 2.5% glutaraldehyde solution (Reagent Grade, Ameresco, BioOhio, USA.) in a centrifuge tube, and kept in a refrigerator at 4°C for electron microscopy.

At least three rats from each group were used for the sample preparation. After the ascending aorta was infused with physiological saline, it was perfused with 4% paraformaldehyde (PFA)/0.1M phosphate buffer saline (PBS, pH 7.4, 4°C, 400–500 mL) until the animal's liver hardened and the tail was stiff, which indicated the completion of the perfusion. The brains were isolated and kept in a 4% PFA/ 0.1M PBS overnight at 4°C (no more than 48 h). The following day, they were transferred to 30% sucrose and dehydrated to a low level. They were routinely dehydrated, cleared, waxed, and paraffin embedded. The first slice was sectioned from 1.0 mm anterior to the anterior ridge in the right half brain for immunohistochemical staining.

### 2.5. Scanning Electron Microscopy (SEM)

The rat brain striatum tissues were rapidly dissected, mounted on toothpicks at resting length, and placed in 2.5% glutaraldehyde in PBS for overnight fixation at 4°C. Postfixation was performed in aqueous 1% osmium tetroxide for 60 min. Specimens were dehydrated with ethanol sequentially 30%, 50%, 70%, 80%, 90%, and 100% ethanol (twice). Ethanol was removed and a 1:1 mixture of isoamyl acetate and ethanol was added, and they were mixed by shaking 10-20 min, and the mixture was discarded and then slowly infiltrated with epoxy resins (Embed 812/Araldite, Electron Microscopy Sciences, Hatfield, PA, USA.), embedded, and then heated overnight at 60°C. For SEM, the specimens were critical point dried, mounted on aluminum stubs, sputtered with a gold coat, and examined under a JEOL 6335F field emission gun scanning electron microscope.

### 2.6. Immunohistochemical Staining of TH and *α*-Synuclein

5*μ*m slices were sectioned by 820 Rotary Tissue Slicer (AO Company, American). Supersense™ Immunohistochemistry Detection System Kit (No.BS13278, Bioworld Technology, Inc., St Louis Park, USA.) was used for the localization, characterization, and quantification of TH and *α*-synuclein in the striatum. The primary antibody anti-TH (NO.BS4010, 1:200, Bioworld Technology) and anti-*α*-synuclein (~20 kD NO.BS3429,1:200, Bioworld Technology) were added to each sample 50 *μ*l/slice, and kept in the refrigerator at 4°C overnight. The secondary antibody 50*μ*l/slice of Goat anti-Rabbit IgG (H&L)-HRP (1:2000, NO.BS13278, Bioworld Technology) was added dropwise to each sample. Then the routine steps were diaminobenzidine staining, hematoxylin and eosin restaining, dehydration with gradient alcohol, and clearing of tissue sections with xylene.

The morphometric analysis was performed using the Image-Pro Plus (IPP) Version 6.0 software (Media Cybernetics, Inc., Rockville, USA.). Five images were taken using Compix Simple PCI (Compix, Inc., Imaging Systems, Pennsylvania, USA.). Five fields were randomly selected under each microscope (×200) for analysis. The immunohistochemically positive anti-TH and anti-*α*-synuclein were stained yellow or brown by 3,3-diaminobenzidine tetrahydrochloride (DAB) (Zhongshan Biotechnology Co., Ltd., Beijing, China). Their integrated optical density (IOD) was evaluated using the area of interest method in Image-Pro Plus Version 6.0 (IPP 6.0).

### 2.7. Determination of miRNA Genomics

#### 2.7.1. miRCURY LNA miRNA Array

The seventh generation of miRCURY LNA miRNA array (v.18.0) (Exiqon, Denmark) contained 3100 capture probes, covering all human, mouse, and rat miRNAs annotated in miRBase 18.0, and all viral miRNAs related to these species [23]. This array also contained capture probes for 25 miRPlus human miRNAs. These were proprietary miRNAs not found in miRBase.

#### 2.7.2. RNA Extraction

About 60 mg of rat striatum was used extracting total RNA with TRIzol reagent (Invitrogen, Carlsbad, California, USA.) and miRNeasy mini kit (Qiagen, Germantown, MD, USA.) according to the manufacturer's protocol, which efficiently recovered all RNA species, including miRNAs. The RNA quality and quantity were measured using a NanoDrop spectrophotometer (ND-1000, NanoDrop Technologies, Wilmington, DE, USA.), and RNA integrity was determined using gel electrophoresis.

#### 2.7.3. miRNA Array

After RNA isolation from the samples, the miRCURY Hy3/Hy5 Power labeling kit (Exiqon, Vedbaek, Denmark) was used according to the manufacturer's guideline for miRNA labeling. One microgram of each sample was 3'-end-labeled with a Hy3 fluorescent label using T4 RNA ligase. After the labeling procedure, the Hy3-labeled samples were hybridized on the miRCURY LNA Array (v.18.0) (Exiqon, Exiqon A/S, Skelstedet 16, 2950 Vedbaek, Denmark) according to the array manual. The 12-Bay Hybridization System (Nimblegen Systems, Inc., WI, USA) was used. Then, the slides were scanned using the Axon GenePix 4000B microarray scanner (Axon Instruments, CA, USA).

#### 2.7.4. miRNA Array Scanning and Data Analysis

Scanned images were then imported into the GenePix Pro 6.0 software (Axon Instruments Inc., Union City, CA, USA) for grid alignment and data extraction. Replicated miRNAs were averaged, and miRNAs with intensities ≥30 in all samples were chosen for calculating the normalization factor. Expressed data were normalized using the median normalization. After normalization, differentially expressed miRNAs were identified using fold change filtering. Finally, hierarchical clustering was performed using the Institute for Genomic Research (TIGR) Multiple Experiment Viewer (MEV) software (v4.6) to show distinguishable miRNA expression profiling among samples. The statistical analysis was performed using the Bioconductor DESeq package (http://www.bioconductor.org/) to detect differential expression levels of miRNAs [24].

#### 2.7.5. Bioinformatics Analysis of miRNAs: Target Prediction and Functional Analysis

All miRNA target data were analyzed using the Ingenuity Pathway Analysis tool and the target prediction database in Target Scan. Three aspects of molecular function, biological process, and cell composition were obtained through the Gene Ontology analysis.

The miRDB (http://mirdb.org/miRDB/) database was used to predict the target of the differentially expressed known miRNAs with a predicted score ≥60. Functional annotation of each target gene was performed using the DAVID (the database for annotation, visualization, and integrated discovery) (http://david.abcc.ncifcrf.gov/) bioinformatics resource (version 6.7), including an integrated biological knowledge base and analysis tools. Biological significance was systematically extracted from large gene/protein families. The information about metabolism, genetic information processing, environmental information processing, cellular processes, and human diseases can be obtained from the database of the Kyoto Encyclopedia of Genes and Genomes (KEGG) website.

### 2.8. Real-Time Fluorescence Quantitative Polymerase Chain Reaction of miRNAs and mRNAs

Real-time fluorescence quantitative (q) polymerase chain reaction (qPCR) was performed as previously described [25]. At least three samples were randomly assigned to each group, and qPCR was performed on each of the samples (Table 1). The data regarding the amplification curves and melting curves of quantitative real-time PCR and cycle threshold (Ct) values of each gene mRNA were recorded. The mRNA expression of the target gene was quantitatively determined using the method of 2-delta Ct as the internal reference.

### 2.9. Statistical Analysis

Data were presented as mean ± SEM. Statistical analysis was performed using predictive analytics software statistics 16.0 (SPSS Inc., Chicago, IL, USA). The experimental groups were compared using ANOVA with repeated measures on the behavior test. A Tukey post-hoc test was used to follow up with significant ANOVA results. Cohn's D was used to examine the effect size. The statistical significance of the effects of the experimental treatment was determined by comparing the areas under the curve (*P* < 0.05, Student's* t*-test).

## 3. Results

### 3.1. Behavioral Test on Rat PD Model

The successful establishment of the 6-OHDA-induced PD model was confirmed by the rotation test using an intraperitoneal (IP) injection of apomorphine hydrochloride [13, 14]. Four rats in SED-PD and six rats in EX-PD were excluded after the first rotation test (less 4 rotations).  Table 2 shows that the number of rotations in the EX-PD group was significantly reduced but not statistically significant compared to the SED-PD group in the first and the second week after surgery (*P* > 0.05).

### 3.2. Scanning Electron Microscopy of Rat Striatum in the PD Model

**Figure 1:**
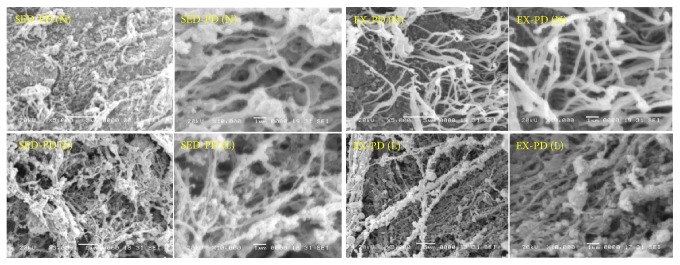
*Scanning electron microscopy images of the striatum of the rat model of PD*. SED-PD, the sedentary control PD group; EX-PD, the aerobic exercise PD group; no-lesion side (N) and lesion side (L).

As shown in Figure 1, the striatum showed fewer neuro-villi and the arrangement was sparse and cluttered in the no-lesion side of SED-PD at 5000-fold magnification. The image at 10,000-fold magnification revealed the neuronal villi grown in a scattered pattern with no entangled plexus and more nodular synaptic junctions on or between the neuronal villi. The image at 3000-fold magnification revealed atrophy of the neuronal villi in the lesion side of SED-PD. A large number of neurites and axons were seen, which were unclear and resembled a scum-like structure.

Figure 1 shows a larger number of nerve villi in the striatum arranged neatly in bundles. Many nodules of synaptic junctions were observed on or between the neuronal villi at 5000-fold magnification in the no-lesion side of EX-PD. Atrophy of the neuronal villi and a large number of neurites and axons were observed in the striatum at 3000-fold magnification in the lesion side of EX-PD. The neurites and axons were entangled in bundles and neatly arranged. Many neuronal villi were observed to be entangled with the neurites and axons. The image at 10,000-fold magnification revealed that the neurites and axons were thick, and still more nodular synaptic connections were found above or between neuronal villi. Our results showed the neurons, axons, and neuronal villi of the striatum were clearly and tightly arranged, and neurons and axons were significantly larger in the EX-PD group compared to the SED-PD group.

### 3.3. TH and *α*-Synuclein Expression in the Rat Striatum

**Figure 2:**
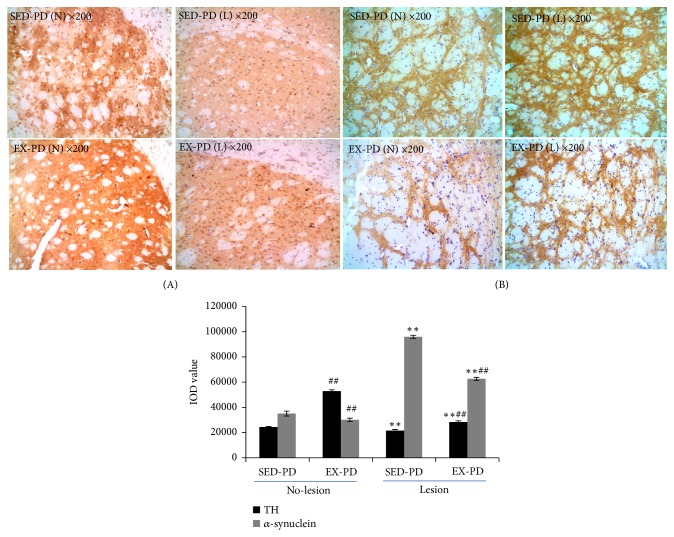
*TH and α-synuclein expression in the striatum of the PD rat model*. (A) Microscopic image of TH; (B). Microscopic image of *α*-synuclein; SED-PD is the sedentary control PD group, EX-PD is the aerobic exercise PD group; ^*∗∗*^*Ρ* < 0.01, comparing between SED-PD and EX-PD; ^##^*Ρ* < 0.01, comparing between no-lesion side (N) and lesion side (L), n=4-6; no-lesion side (N) and lesion side (L); TH, tyrosine hydroxylase.

*α*-Synuclein protein aggregation was the major structural component of the intracellular protein inclusions termed Lewy bodies that define the hallmark of PD [26]. And TH expression decreased in the striatum is another marker involved in the pathology of PD [27]. Immunohistochemical staining of TH and *α*-synuclein protein was abundantly expressed in the cytoplasm of the striatum and sparingly expressed in the stroma as a brownish color, with a small number of nuclei also tan and normal nuclei stained blue. TH and *α*-synuclein positively stained area were large, and the protein stained dark in color in the striatum in SED-PD and EX-PD. Compared with the no-lesion side, TH expression was decreased significantly in the lesion side in both SED-PD and EX-PD groups (*P* < 0.01); but compared to the SED-PD group, TH expression was significantly increased in EX-PD preexecuted with regular aerobic exercise (*P* < 0.01) (Figure 2). Compared to the no-lesion side, *α*-synuclein expression in the lesion side significantly increased in the striatum in SED-PD and EX-PD (*P* < 0.01). Intriguingly, compared to the SED-PD group, *α*-synuclein was significantly decreased in EX-PD (*P* < 0.01) (Figure 2).

### 3.4. Effect of Differentially Expressed miRNAs in the Striatum of the Rat Model of PD

Based on the preimplementation of regular aerobic exercise and the impact of miRNAs on PD-related expression profiles, this study standardized and screened the unreliable data and screened 11 miRNAs upregulated by regular aerobic exercise to two-fold or higher (multifold) in the striatum of the rat model of PD: miR-1-3p, miR-34c-3p, miR-31a-3p, miR-499-3p, miR-200b-5p, miR-465-3p, miR-190a-3p, miR-146a-3p, miR-2985, miR-377-3p, and miR-3557-3p (Table 3). Further, 10 miRNAs were downregulated by twofold or more: miR-136-5p, miR-138-5p, miR-324-5p, let-7e-3p, miR-770-5p, miR-134-5p, miR-935, miR-653-3p, miR-331-5p, and miR-539-3p (Table 3).

The bioinformatics analysis was performed on the aforementioned 21 miRNAs: target prediction and functional analysis. The subcellular localization, the role of target genes in known diseases, and pathway screening of the target gene pairs were investigated according to the expression level. The reliable miRNA-mRNA interaction network was selected from the target database by predicting transcription factors and molecular activity. miR-3557 and miR-324 were worth further exploration in this experiment.

### 3.5. Differential Screening of miR-3557 and miR-324

#### 3.5.1. Gene Ontology (GO) and KEGG Biological Pathway Analysis

According to the GO bioinformatics analysis, miR-3557 and miR-324 had molecular functions. The analysis showed that 218 targeted genes were involved in energy metabolism, 42 in metabolic regulation, 44 in developmental processes, 75 in biological regulation, and 64 in stimulus response. Gene-related biological functions were regulation of sensory and pain perception, integration of regulatory signaling pathways, phosphatidylinositol metabolic processes, positive regulation of biological processes, and positive regulation of transmembrane transporter activity.

miR-3557-3p is closely related to CaMKV, nuclear heterogeneous riboproteins; and miR-324 is closely related to Vdac1. The KEGG biological pathway analysis predicts possible enrichment of biological pathways and classifies the results with statistical significance. These statistically significant biological pathways include the calcium signaling pathway (rno04020), neuroactive ligand-receptor interaction (rno04080), DNA replication (rno03030), extracellular matrix- (ECM-) receptor interaction (rno04512), amphetamine addiction (rno05031), glycosylphosphatidylinositol- (GPI-) anchor biosynthesis (rno00563), B cell receptor signaling pathway (rno04662), endocytosis (rno04144), drug metabolism-cytochrome P450 (rno00982), and nucleotide excision repair (rno03420). These biological signaling pathways may have a statistically significant relationship with PD.

#### 3.5.2. Validation of miR-3557 and miR-324

**Figure 3:**
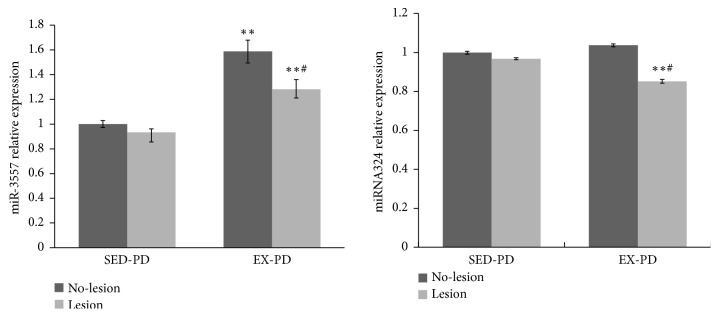
*MiR-3557 and miR-324 expression by real-time quantitative PCR assay*. ^*∗∗*^*P* < 0.01, comparing between SED-PD and EX-PD; ^#^*P* < 0.05, comparing between no-lesion side (N) and lesion side (L); SED-PD is the sedentary control PD group, EX-PD is the aerobic exercise PD group.

The analysis of target genes showed that miR-3557 was upregulated and miR-324 was downregulated. Real-time quantitative PCR was used to verify the consistency of the results of miRNA expression and microarray. The expression of miR-3557 was significantly upregulated in the EX-PD group compared to the SED-PD group (*P* < 0.01) (Figure 3). Further, the expression of miR-3557 was significantly downregulated in Lesion side compared to no-lesion side in EX-PD (*P* < 0.05).

Figure 3 also shows that the expression of miR-324 was significantly downregulated in the lesion side of EX-PD group compared to the SED-PD group (*P* < 0.01), but the expression of miR-324 was slightly downregulated in the lesion side compared to the no-lesion side in SED-PD. The expression of miR-324 was significantly downregulated in the lesion side compared to the no-lesion side in EX-PD (*P* < 0.05).

### 3.6. Effect of CaMKs/mTOR Signaling Pathway in the Striatum of the Rat Model of PD

**Figure 4:**
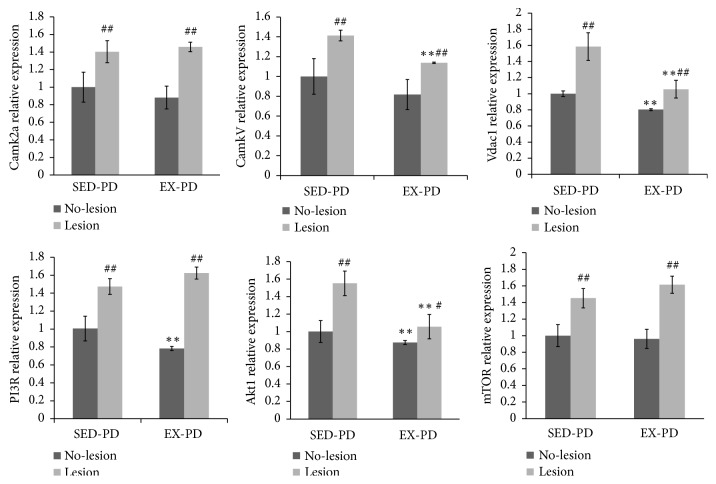
*Expression levels of CaMKs/mTOR signal related mRNA by real-time quantitative PCR assay*. ^*∗∗*^*P* < 0.01, comparing between SED-PD and EX-PD; ^#^*P* < 0.05, ^##^*P* < 0.01, comparing between no-lesion side (N) and lesion side (L); SED-PD is the sedentary control PD group, EX-PD is the aerobic exercise PD group.

Figure 4 shows that* Camk2α, CamkV,* and* Vdac1* mRNA expression was significantly upregulated in the lesion side compared to the no-lesion side in SED-PD and EX-PD (*P* < 0.01).* CamkV* and* Vdac1* mRNA expression was downregulated in the lesion side of EX-PD compared with the SED-PD (*P* < 0.01), but* Camk2α* was slightly upregulated. Intriguingly,* Camk2α* and* CamkV* mRNA expression was also downregulated in the no-lesion side of EX-PD compared with the SED-PD, and* Vdac1* significantly downregulated (*P* < 0.01).

The real-time quantitative PCR analysis showed that* PI3R*,* Akt*, and* mTOR* mRNA expression (Figure 4) was significantly upregulated in the lesion side compared to the no-lesion side in SED-PD (*P* < 0.01) and EX-PD group except* Akt1* (*P* < 0.01).* PI3R*,* Akt,* and* mTOR* mRNA expression was downregulated in the no-lesion side of EX-PD compared to SED-PD. However,* PI3R* and* mTOR* mRNA expression was slightly upregulated in the lesion side of EX-PD group compared to the SED-PD group, but the expression of* Akt1* was significantly downregulated (*P* < 0.01). In summary, regular aerobic exercise activated the effect of PI3R and mTOR mRNA expression.

### 3.7. Effect of Downstream Molecules in the Rat Model of PD

**Figure 5:**
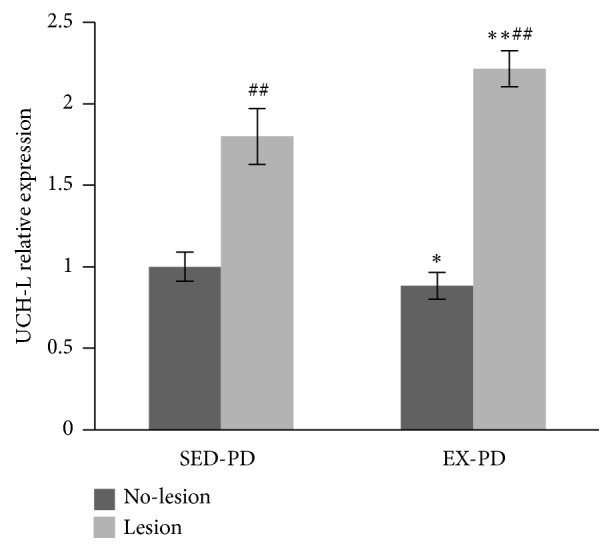
*Expression levels of UCH-L 1 mRNA by real-time quantitative PCR assay*. ^*∗*^*P* < 0.05, ^*∗∗*^*P* < 0.01, comparing between SED-PD and EX-PD; ^##^*P* < 0.01, comparing between no-lesion side (N) and lesion side (L); SED-PD is the sedentary control PD group, EX-PD is the aerobic exercise PD group.

U-ubiquitin C-terminal hydrolase 1 (*UCH-L1*) mRNA expression (Figure 5) was significantly upregulated in the lesion side compared to the no-lesion side in SED-PD and EX-PD (*P* < 0.01). However,* UCH-L1* mRNA expression was significantly downregulated in the no-lesion side in EX-PD compared to SED-PD (*P* < 0.05). But, intriguingly,* UCH-L1* mRNA expression was upregulated in the lesion side of the EX-PD group compared to the SED-PD group (*P* < 0.01).

## 4. Discussion

Exercising both before and after 6-OHDA could maximize and improve the possibility of motor function by exercise-induced improvements in PD rats [15, 28, 29]. Increasing aerobic treadmill exercise before unilateral intrastriatal 6-OHDA injection has been shown to rescue motor deficits, measured by the apomorphine-induced rotational asymmetry in rats [13, 14]. In this study, regular aerobic exercise before unilateral intrastriatal 6-OHDA injection improved the PD rat's behavior and saved the structure of striatum in PD rat from scanning electron microscopy observation. Regular aerobic exercise significantly increased TH expression and decreased *α*-synuclein expression. Our experimental results provided evidence that regular aerobic exercise alleviated the neurodegenerative process of PD lesions. The specific mechanism of exercise effects is still unclear.

MiRNAs act on many targeted genes involved in the development of neurological diseases or neurodegenerative diseases. Hua et al. [30] discovered more than 30 specific and highly expressed miRNAs in rat neural tissue and speculated that these miRNAs with specific high expression in rat neural tissue might help in the diagnosis and treatment of neurological diseases. Rogaev et al. [31] found that miRNAs were involved in the occurrence of many neuropsychiatric disorders, especially those associated with developmental abnormalities. Yet, no known study has examined how the miRNAs (examined via miRNAs profiling) might differ between PD animals who* regularly* exercise interventions and PD animals who are* habitually* sedentary. We further found that 11 miRNAs, such as miR-3557-3p, were upregulated by twofold or more in the striatum of rats with PD on the preimplementation of regular aerobic exercise. And we also found 10 miRNAs, such as miR-324-5p, were downregulated by twofold or more. Feng et al. [32] found upregulation of rno-miR-3557-5p could be seen as biomarkers of prognosis in clinical therapy of heart failure. Diaz et al. [33] found that the overexpression of miR-324-3p promoted the dysregulation of the expression of genes involved in cell death and apoptosis, cAMP and Ca^2+^ signaling, cell stress, and metabolism. In summary, the subcellular localization, the role of target genes in known diseases, and pathway screening of the target gene pairs were investigated according to the expression level in this study. Also, the most reliable miRNA-mRNA interaction network was screened by predicting transcription factors and molecular activity. The results showed that miR-3557 and miR-324 deserve further exploration.

The bioinformatics analysis showed that miR-3557-3p was closely related to CaMKV, nuclear heterogeneous riboproteins, and macro-related proteins; miR-324 was closely related to Vdac1. The KEGG biological pathway analysis predicted the possible enrichment of biological pathways, including calcium signaling pathways, stimulated nervous system interactions with receptors, and DNA replication pathways. CaMKs is highly expressed in neurons and has a specific subcellular localization. It converts intracellularly elevated calcium signals into a range of target proteins, including ion channels and transcriptional activators, and regulates synaptic transmissions related to the downstream signaling pathway [34, 35]. Firstly, CaMKs regulate synaptic plasticity and neurotransmitter release. Zhu et al. [36] showed that CaMKs could activate TH. TH is a rate-limiting enzyme that catalyzes tyrosine to form dopa. After dopa formation, it can be further transformed into neurotransmitters norepinephrine and DA. Phosphorylated TH can increase the synthesis of catecholamines. CaMKs can also directly promote DA release. Secondly, CaMKs play a vital role in the Ca^2+^/CaM signaling system, different concentrations and subcellular regions of Ca^2+^ activate a range of substrates of CaMK2, or PP1 (Ser/Thr phosphatase), which appears to be critical in controlling CaMKs-dependent neuronal signaling [37].

CaMKs pathways are involved in many upstream signaling pathways, such as growth factor signaling, PI3K/Akt, and calcium signaling pathway [38]. mTOR is a serine/threonine protein kinase that serves as the site of energy-sensitive pathways for PI3K/Akt and serine/threonine kinase II [39]. mTOR not only regulates cell proliferation and survival but also participates in membrane transport and protein degradation, especially in the regulation of protein translation levels. Recently, mTOR has been shown to promote autophagy, remove abnormal proteins such as amyloid polypeptide in AD and mutated *α*-synuclein in familial PD, and thus treat related diseases [40]. This provides a new treatment strategy for preventing and treating geriatric diseases. Our results showed that PI3R and mTOR mRNA expression was slightly upregulated in the lesion side in EX-PD compared with the SED-PD group and showed that regular aerobic exercise improved the effect size of PI3R and mTOR mRNA expression. This indicated that preregular aerobic exercise induced the activation of mTOR signaling pathway and regulating to remove abnormal proteins mutated *α*-synuclein in the PD model rat striatum. Ankovic [41] reported that mTOR had the potential to treat PD. Pan et al. [42] found that the autophagy inducer rapamycin, specifically targeting the autophagy-gated mTOR, could alleviate this phenomenon. Inhibition of autophagy exacerbated the accumulation of *α*-synuclein leading to cell death, rapamycin-induced the degradation of *α*-synuclein and cell survival.

The secretion and expression of UCH-L1 could be involved in the pathology of Parkinson's disease. Lowe et al. [43] reported mutations in *α*-synuclein and UCH-L1 genes in familial PD and the presence of these proteins in Lewy bodies of sporadic PD, indicating errors in the production of these proteins during folding and degradation, which could cause nerve cell degeneration. Mandel et al. [44] found the increase in the UCH-L1 level is favorable for DA neurons to clear abnormal proteins, delay the accumulation of abnormal proteins, and inhibit the formation of Lewy bodies. Manago et al. [45] found that UCH-L1 increased ATP-induced currents were dependent on cAMP-dependent protein kinase (PKA) and CaMK2. Zhang et al. [46] reported that conventional protein kinase C (cPKC) could regulate oxidative stress and apoptosis through UCH-L1; inhibiting UCH-L1 expression can negatively regulate autophagy through the extracellular signal-regulated kinase (ERK)-mTOR pathway. We found the expression of UCH-L1 mRNA expression was upregulated in the lesion side of EX-PD group compared with SED-PD group. It is shown that preregular aerobic exercise also activated the expression of UCH-L1 in the PD lesions. Our results indicated that regular aerobic exercise alleviated the neurodegenerative process of PD lesions by improving UCH-L1 level, which might be the downstream of CaMKs or mTOR signal regulation. Kanthasamy et al. [47] found that ROS and oxidative damage were involved in the disease process of PD. UCH-L1 could effectively remove ROS and protect neurons from oxidative damage. Therefore, UCH-L1 reflects the self-protection mechanism of neurons in experimental animals. Whether the change in the UCH-L1 level can inhibit the occurrence or development of PD lesions on aerobic exercise may be worth further exploring.

## 5. Conclusions

The adaptive mechanism of regular aerobic exercise delaying the development of neurodegenerative diseases and lesions was that miR-3557/324 was activated to regulate one of its targets CaMKs signaling pathways. CaMKs coordinated with mTOR pathway-related gene expression and improved UCH-L1 level to favor for tyrosine hydroxylase and clear abnormal *α*-synuclein, thereby delaying neurodegeneration or improving the pathogenesis of PD lesions. This study provided important evidence and theoretical guidance for motor rehabilitation.

## Figures and Tables

**Table 1:** Real-time fluorescence quantitative PCR primers for target miRNAs and mRNAs.

Gene	Sequence (5′—3′)
miRNA reference primers	GeneCopoeia, Inc, USA. Catalog#: RmiRQP9003
rno-miR-3557-3p	GeneCopoeia, Inc, USA. Catalog#: RmiRQP1817
rno-miR-324-5p	GeneCopoeia, Inc, USA. Catalog#: RmiRQP0412
*β*-actin	F: GAGATTACTGCTCTGGCTCCTA R: GGACTCATCGTACTCCTGCTTG
*Camk 2a*	GeneCopoeia, Inc, USA. Catalog#: RQP049239
*Camk V*	GeneCopoeia, Inc, USA. Catalog#: RQP050747
*Vdac1*	GeneCopoeia, Inc, USA. Catalog#: RQP051105
*Itpr1(PI3R)*	GeneCopoeia, Inc, USA. Catalog#: RQP045271
*Akt1*	GeneCopoeia, Inc, USA. Catalog#: RQP051482
*mTOR*	GeneCopoeia, Inc, USA. Catalog#: RQP050125
*UCH-L1*	GeneCopoeia, Inc, USA. Catalog#: RQP049756

**Table 2:** Rotation experiment in the PD model (unit: number of turns).

Groups	Injection of apomorphine in the first postoperative week			Injection of apomorphine in the second postoperative week		
	The first-10min	The second-10min	The third-10min	The first-10min	The second-10min	The third-10min
SED-PD	8.00±2.00	7.29±1.30	7.29±1.29	7.75±1.49	8.50±1.05	8.00±1.32
EX-PD	5.63±1.55	6.20±1.19	6.25±1.58	7±1.40	7.6±1.08	6.8±1.16

SED-PD is the sedentary control PD group; EX-PD is the aerobic exercise PD group

**Table 3:** Differential miRNAs in the striatum of the rat model of PD on regular aerobic exercise.

ID	Gene	log2 fold change	P-value
More than two-fold upregulated miRNAs			
14294	rno-miR-1-3p	6.5784	P <0.01
42767	rno-miR-34c-3p	3.6394	P <0.05
46320	rno-miR-31a-3p	4.3727	P <0.01
145692	rno-miR-499-3p	10.8036	P <0.01
145974	rno-miR-200b-5p	7.3004	P <0.01
148207	rno-miR-465-3p	2.2084	P <0.05
148330	rno-miR-190a-3p	3.0857	P <0.05
148365	rno-miR-146a-3p	3.4221	P <0.05
148439	rno-miR-2985	3.9897	P <0.05
148549	rno-miR-377-3p	3.1776	P <0.05
148581	rno-miR-3557-3p	21.2246	P <0.01
More than two-fold downregulated miRNAs			
10943	rno-miR-136-5p	0.5312	P <0.01
13140	rno-miR-138-5p	0.5830	P <0.01
42477	rno-miR-324-5p	0.4613	P <0.05
42743	rno-let-7e-3p	0.4045	P <0.01
42817	rno-miR-770-5p	0.4294	P <0.01
42942	rno-miR-134-5p	0.5081	P <0.05
145742	rno-miR-935	0.4532	P <0.05
148174	rno-miR-653-3p	0.1252	P <0.01
148385	rno-miR-331-5p	0.3686	P <0.05
148507	rno-miR-539-3p	0.3345	P <0.05

## Data Availability

The data used to support the findings of this study are available from the corresponding author upon request.
